# Multiple Plattenepithelkarzinome auf einer linearen Porokeratose

**DOI:** 10.1007/s00105-025-05565-2

**Published:** 2025-08-19

**Authors:** Dorothee A. Busch, Carola Berking, Stefan Schliep, Cornelia Erfurt-Berge

**Affiliations:** 1 Hautklinik, Uniklinikum Erlangen, Friedrich-Alexander-Universität Erlangen-Nürnberg, Ulmenweg 18, 91054 Erlangen, Deutschland; 2 Comprehensive Cancer Center Erlangen-European Metropolitan Area of Nuremberg (CC ER-EMN), 91054 Erlangen, Deutschland

**Keywords:** Genetisch bedingte Hautkrankheiten, Fallbericht, Kutane Neoplasmen, Maligne Neoplasmen, Screening, Skin diseases, genetic, Case report, Cutaneous neoplasms, Malignant neoplasms, Screening

## Abstract

Dieser Fallbericht beschreibt einen 58-jährigen Patienten mit linearer Porokeratose, auf deren Basis sich kutane Plattenepithelkarzinome entwickelten. Porokeratose ist eine seltene Keratinisierungsstörung, die mit einem Risiko für eine maligne Transformation einhergeht. Der Patient stellte sich zunächst mit wiederkehrenden Erosionen und Ulzerationen vor. Nachdem er zunächst sehr zurückhaltend war, ärztliche Hilfe in Anspruch zu nehmen, willigte er schließlich in die Entnahme von Probebiopsien ein. Diese bestätigten Plattenepithelkarzinome in mehreren Läsionen sowie ein Bowen-Karzinom. Diese wurden erfolgreich exzidiert, und der Patient entschied sich postoperativ für eine sekundäre Wundheilung. Aufgrund ihres Potenzials zur malignen Transformation erfordern Porokeratosen oft eine sorgfältige klinische Überwachung. Sie sind typischerweise durch hyperkeratotische Läsionen gekennzeichnet, die im Laufe der Zeit fortschreiten können. Obwohl es verschiedene Behandlungsmöglichkeiten der Porokeratose gibt, ist deren langfristige Wirksamkeit begrenzt. Dieser Fall unterstreicht die Bedeutung von frühzeitigen Biopsien bei nicht heilenden Wunden sowie die Notwendigkeit der Edukation von Patienten und regelmäßiger Nachuntersuchungen, um das Hautkrebsrisiko zu reduzieren.

## Abstract (en)

This case report discusses a 58-year-old patient with linear porokeratosis who subsequently developed cutaneous squamous cell carcinoma. Porokeratosis is a rare keratinisation disorder which carries a risk of malignant transformation. The patient initially presented with recurrent erosions and ulcerations. After initial reluctance of the patient to seek medical help, biopsies confirmed squamous cell carcinoma in several lesions. These were successfully excised and the patient opted for secondary wound healing. Due to its potential for malignancy, porokeratosis often requires diligent monitoring. It typically features hyperkeratotic lesions that can progress over time. Although there are various treatment options for porokeratosis, long-term efficacy is limited. This case emphasises the importance of early biopsy in chronic, nonhealing wounds, as well as the need for patient education and regular follow-up to effectively manage the risk of malignancy.

## Anamnese

Ein 58-jähriger Patient mit Fitz-Patrick-Hauttyp II stellte sich mit seit mehreren Monaten rezidivierenden Erosionen und Ulzerationen an seinem linken Bein vor. Die Haut war sehr fragil, riss immer wieder ein und blutete, sodass multiple, nur langsam abheilende Erosionen an seinem Unterschenkel entstanden. Der Patient berichtete, seit dem Alter von 6 Monaten zunehmend hyperkeratotische Papeln und Plaques in linearer Anordnung an seinem linken Bein entwickelt zu haben. Im Alter von 20 Jahren waren zusätzlich ringförmige Papeln und Plaques an anderen Körperstellen aufgetreten. Die lineare Porokeratose war in der Kindheit klinisch und histologisch diagnostiziert worden. Zudem besteht eine disseminierte, superfizielle aktinische Porokeratose an den Extremitäten und vereinzelt am Stamm. Auch seine Mutter und 2 seiner Geschwister hatten ähnliche disseminierte Hautveränderungen. Bis zu seinem 18. Lebensjahr wurden 12 chirurgische Exzisionen der linearen Läsionen an seinem linken Bein durchgeführt, was für den Patienten eine emotional traumatische Erfahrung war. Er suchte daher über einen längeren Zeitraum keine ärztliche Hilfe auf und lehnte bei seiner ersten Vorstellung die Entnahme einer Probebiopsie aus der krustig-erosiven Hautveränderungen ab und stellte sich erst mehrere Jahre später erneut vor. Die erosiven Areale wurden entsprechend zunächst mit antimikrobiellen Wundauflagen behandelt.

## Untersuchung

**Abb. 1:**
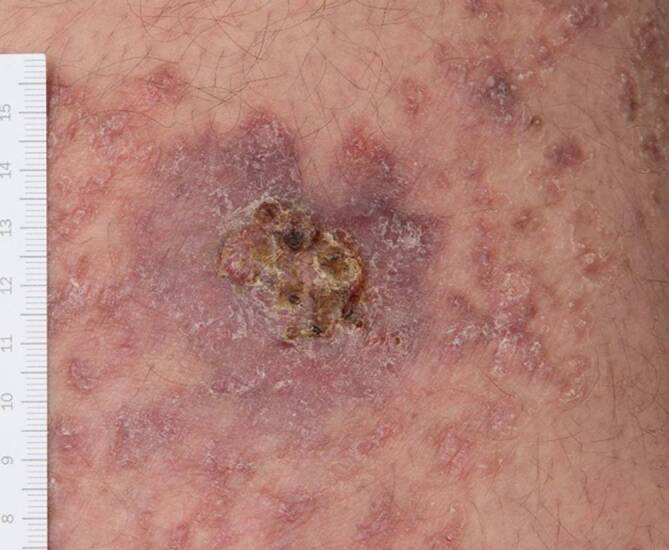
Verkrustete Ulzeration, Porokeratose in der umgebenden Haut

Entlang der linken unteren Extremität befanden sich asymptomatische, linear angeordnete erythematöse, hyperkeratotische und teilweise konfluierende Papeln mit Erosionen und Krusten. Am linken Oberschenkel zeigte sich eine knotige, verkrustete, erosive Plaque (Abb. 1). Darüber hinaus waren an beiden Armen und am rechten Bein multiple erythematöse runde Makulae mit schuppigen Rändern zu sehen.

## Diagnostik

**Abb. 2:**
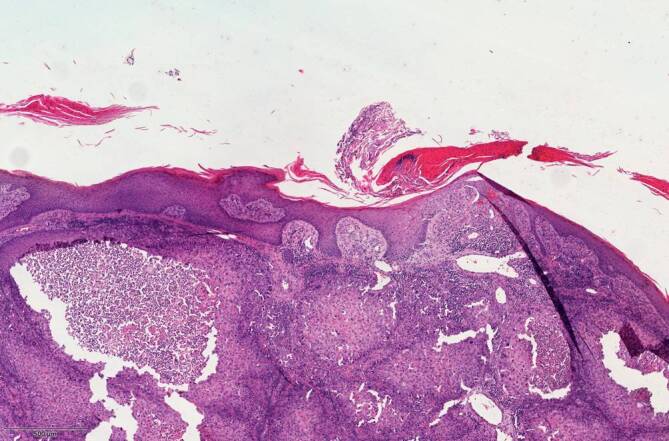
Histologisches Bild eines der beschriebenen Plattenepithelkarzinome mit Anteilen einer kornoiden Lamelle

**Abb. 3:**
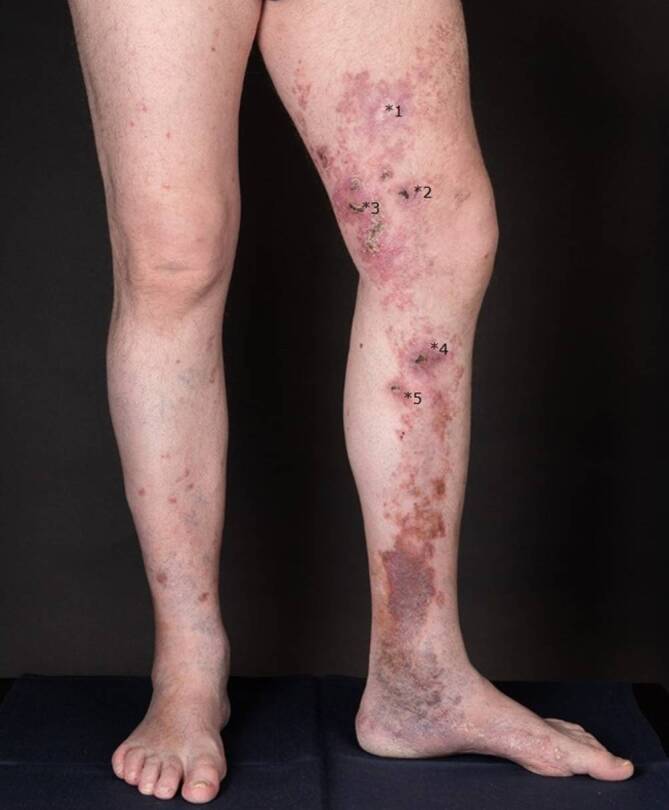
Biopsierte Hautveränderungen: *1 postoperative Narbe nach Exzision eines Plattenepithelkarzinoms, Tumordicke 4 mm, *2 reaktive epidermale Hyperplasie und Narbengewebe ohne Hinweis auf Malignität, *3 Plattenepithelkarzinom, Tumordicke 0,8 mm mit aktinischer Keratose in der Umgebungshaut, *4 ulzeriertes Plattenepithelkarzinom, Tumordicke 1,1 mm, *5 Narbengewebe ohne Hinweis auf Malignität

**Abb. 4:**
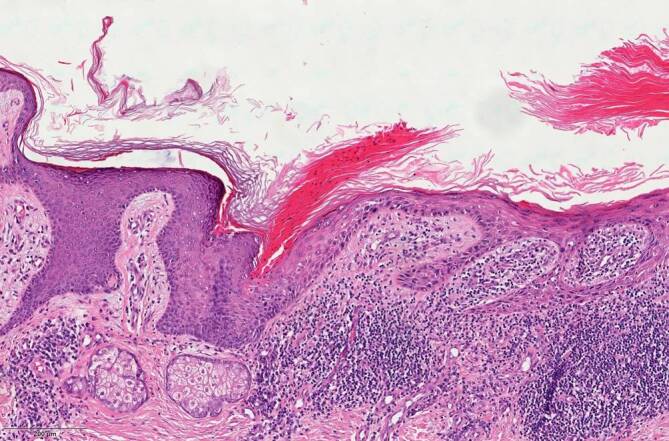
Histologisches Bild einer kornoiden Lamelle sowie Übergang zur epithelialen Dysplasie

Differenzialdiagnostisch kämen bei den Hautveränderungen am linken Bein neben der bekannten Porokeratose ebenso ein inflammatorischer verruköser epidermaler Nävus, ein linearer Lichen ruber sowie eine Incontinentia pigmentii in Betracht. Bezüglich der krustigen Knoten und Plaques bestand der Verdacht auf Hauttumoren. Nach intensiver Edukation und Aufklärung willigte der Patient schließlich in Probebiopsien aus den mittlerweile 6 verdächtigen Läsionen am linken Ober- und Unterschenkel ein. Die prominenteste ulzerierte Plaque am linken Oberschenkel wurde histologisch als ulzeriertes Bowen-Karzinom bestätigt und mikrographisch kontrolliert exzidiert. Aufgrund anamnestisch bekannter Wundheilungsstörungen entschied sich der Patient für eine Sekundärheilung ohne Hauttransplantation, da er eine Hautnaht aufgrund negativer Vorerfahrungen durch seine Porokeratose ablehnte. Weitere Biopsien der darunterliegenden Plaques zeigten an 2 Stellen kutane Plattenepithelkarzinome (Abb. 2) und aktinische Keratosen in der umgebenden Haut (Abb. 3). Alle Karzinome konnten vollständig und mikrographisch kontrolliert exzidiert werden. Die histopathologischen Untersuchungen bestätigten zudem die Porokeratose mit klassischen kornoiden Lamellen in der umgebenden Haut (Abb. 4). Immunhistochemisch waren Cytokeratin 5 und Cytokeratin 6 stark exprimiert.

## Therapie und Verlauf

Postoperativ entschied sich der Patient erneut für eine sekundäre Wundheilung. Die Ultraschalluntersuchung der Leistenlymphknoten ergab keinen Hinweis auf eine Metastasierung. Bei den regelmäßigen Nachuntersuchungen zeigten die Wunden eine zufriedenstellende Heilung, während in Hautbereichen, an denen klebende Verbandsmaterialien verwendet wurden, oberflächliche Erosionen zurückblieben. Es dauerte insgesamt 10 Wochen bis zur vollständigen Abheilung der Wunden. Eine photodynamische Therapie ist geplant, um okkulte präkanzeröse Läsionen zu behandeln, und der Patient ist engmaschig für Ganzkörperinspektionen angebunden.

## Diskussion

Porokeratosen bezeichnet eine Gruppe seltener Keratinisierungsstörungen mit heterogener klinischer Präsentation [7]. Pathogenetisch ist unter anderem der Mevalonat-Weg, der an der Cholesterinsynthese beteiligt ist, betroffen [4]. Cholesterin ist wiederum Bestandteil der extrazellulären Lipidlamellen in der Hornschicht der Epidermis [9]. Neben genetischer Disposition können auch extrinsische Faktoren wie Immunsuppression, mechanisches Trauma, Erreger und UV-Strahlung zur Entstehung einer Porokeratose beitragen [6].

Es existieren verschiedene klinische Varianten, darunter die Porokeratose Mibelli, die lineare Porokeratose und die disseminierte superfizielle aktinische Porokeratose als häufigste Varianten [9]. Wie im Fall unseres Patienten kommt es gelegentlich vor, dass die disseminierte superfizielle aktinische Porokeratose und die lineare Porokeratose gleichzeitig auftreten [4]. Das klinische Erscheinungsbild der Porokeratosen ist breit gefächert, wobei sich typischerweise anuläre oder lineare Makulae oder Plaques mit einem hyperkeratotischen Rand und einem leicht atrophen Zentrum finden [8, 9]. Die Diagnose wird meist klinisch gestellt und kann histologisch gesichert werden. Hier zeigt sich typischerweise die kornoide Lamelle, eine mit Keratin gefüllt epidermale Invagination mit parakeratotischen Zellen [8].

Bisher gibt es keine eindeutigen Behandlungsleitlinien für Porokeratosen [8, 9], und die verfügbaren Therapien sind selten langfristig effektiv. Dennoch existieren zahlreiche chirurgische, systemische und topische Behandlungsansätze [8, 9]. Unbehandelt werden Porokeratoseläsionen höchstwahrscheinlich persistieren [5]. Häufig eingesetzte Behandlungen umfassen topisches Vitamin D, topische und systemische Retinoide, topisches Imiquimod, Kryochirurgie, Laserablation und konventionelle Chirurgie [8]. Ein vielversprechender neuer Ansatz zur Behandlung der Porokeratose ist die topische Anwendung von Cholesterin in Kombination mit Statinen wie Lovastatin, Simvastatin oder Atorvastatin [1, 4].

Aufgrund der chronischen Entzündung der Haut kann in allen Formen der Porokeratose eine maligne Transformation auftreten [8]. Insbesondere bei langer Bestehensdauer der Läsionen, linearer Porokeratose und größeren Läsionen kann eine maligne Transformation auftreten [5, 8].

Wie häufig es zu einer malignen Entartung kommt, ist umstritten. Die meisten Schätzungen basieren auf Literaturübersichten und sind möglicherweise durch Publikationsbias beeinflusst [3]. Eine retrospektive Auswertung von 110 Patienten mit Porokeratose zeigte eine Rate maligner Transformationen von 6,4–16,4 % [3]. Sasson und Krain fanden in einer Übersicht von 281 publizierten Fällen eine Rate von 7,5 % [5]. In einer landesweiten Analyse von über 2000 Patienten mit Porokeratose fanden Inci et al., dass das Risiko, ein Plattenepithelkarzinom zu entwickeln, im Vergleich zur gematchten Kontrollgruppe erhöht war (Hazard Ratio 4,3) [2]. Eine konsequente Therapie der Porokeratose und regelmäßige Ganzkörperinspektionen zur Erkennung von malignen Hautveränderungen oder Vorstufen sind daher indiziert.

## Fazit für die Praxis

- Porokeratosen, insbesondere die lineare Form, tragen das Risiko einer malignen Transformation und der Entwicklung von Hautkrebs, insbesondere Plattenepithelkarzinomen.
- Bei auffälligen Plaques, Knoten und Erosionen mit fehlender Abheilung sollten frühzeitig Probebiopsien entnommen werden.

## Data Availability

Die Daten, die die Ergebnisse dieser Kasuistik unterstützen, sind vom Universitätsklinikum Erlangen, Abteilung für Dermatologie, erhältlich. Die Daten sind bei den Autoren auf angemessene Anfrage mit Genehmigung des Universitätsklinikums Erlangen, Abteilung für Dermatologie, erhältlich.
